# Adipose-Tissue-Derived Mesenchymal Stem Cells Mediate PD-L1 Overexpression in the White Adipose Tissue of Obese Individuals, Resulting in T Cell Dysfunction

**DOI:** 10.3390/cells10102645

**Published:** 2021-10-03

**Authors:** Assia Eljaafari, Julien Pestel, Brigitte Le Magueresse-Battistoni, Stephanie Chanon, Julia Watson, Maud Robert, Emmanuel Disse, Hubert Vidal

**Affiliations:** 1 Inserm U1060, University Claude Bernard Lyon 1, INRAE U1397, 69310 Pierre Bénite, France; julien.pestel@univ-lyon1.fr (J.P.); brigitte.lemagueresse@inserm.fr (B.L.M.-B.); Stephanie.chanon@univ-lyon1.fr (S.C.); Julia.watson@etu.univ-lyon1.fr (J.W.); maud.robert@chu-lyon.fr (M.R.); emmanuel.disse@chu-lyon.fr (E.D.); hubert.vidal@univ-lyon1.fr (H.V.); 2 CarMeN Laboratory, Centre Hospitalier Lyon Sud, 69310 Pierre Bénite, France; 3 Direction des Affaires Médicales, Hospices Civils de Lyon (HCL), 69002 Lyon, France; 4 Bariatric Surgery Department, Edouard Herriot Hospital, 69003 Lyon, France; 5 Endocrinology Department, Lyon Sud, 69310 Pierre Bénite, France

**Keywords:** PD-1/PD-L1 immune checkpoints, T cell dysfunction, adipose-tissue-derived mesenchymal stem cells, white adipose tissue, inflammation, obesity, IFNγ

## Abstract

The PD-L1/PD-1 immune checkpoint axis is the strongest T cell exhaustion inducer. As immune dysfunction occurs during obesity, we analyzed the impact of obesity on PD-L1/PD-1 expression in white adipose tissue (WAT) in mice and in human white adipocytes. We found that PD-L1 was overexpressed in WAT of diet-induced obese mice and was associated with increased expression of PD-1 in visceral but not subcutaneous WAT. Human in vitro cocultures with adipose-tissue-derived mesenchymal stem cells (ASC) and mononuclear cells demonstrated that the presence of ASC harvested from obese WAT (i) enhanced PD-L1 expression as compared with ASC from lean WAT, (ii) decreased Th1 cell cytokine secretion, and (iii) resulted in decreased cytolytic activity towards adipocytes. Moreover, (iv) the implication of PD-L1 in obese ASC-mediated T cell dysfunction was demonstrated through PD-L1 blockade. Finally, (v) conditioned media gathered from these cocultures enhanced PD-L1 expression in freshly differentiated adipocytes, depending on IFNγ. Altogether, our results suggest that PD-L1 is overexpressed in the WAT of obese individuals during IFNγ secretion, leading to T cell dysfunction and notably reduced cytolytic activity. Such a mechanism could shed light on why adipose-tissue-infiltrating viruses, such as SARS-CoV-2, can worsen disease in obese individuals.

## 1. Introduction

Effective responses to intracellular pathogens or tumors are usually dependent on Natural killer (NK) cells and/or T cells, especially CD8+ cytotoxic T cells. NK cell killing can be regulated by killer cell inhibitory receptors (KIR) after being bound to HLA class I molecules [1]. Exhaustion is another way of inhibiting cytotoxic functions in either NK or CD8+ cytolytic T cells (CTL), due to increased protein expression of inhibitory receptors such as PD-1, CTLA-4, TIM-3, LAG-3, or TIGIT on T cells, or NKG2A on NK cells, and their respective ligands PD-L1 or PD-L2, CD 155, CD112, CD113, and HLAG/E on antigen-presenting cells [2]. These molecules are called immune checkpoints and the PD-1/PD-L1 axis is known as the strongest exhaustion inducer [3]. Moreover, tumors have been shown to use this pathway in order to escape antitumor immunity with the help of pro-inflammatory cytokines, particularly IFNγ [4]. Τhe expression of PD-L1 in tumors is now considered a good prognostic factor, due to the great improvement in patient outcome with immune checkpoint inhibitors [5]. A Nobel Price of Medicine was awarded in 2018 to James Allison and Tasuku Honjo for discovering immune checkpoints, which have been shown to have strong beneficial effects in cancer therapy after PD-L1 or PD-1 blockade. Several studies have demonstrated a negative correlation between CD4+ or CD8+ T cell frequencies and the severity of SARS-CoV-2 infection [6,7]. Indeed, total CD3+, CD8+, or CD4+ T cell frequencies below 800, 300, or 400/μL, respectively, were negatively associated with survival of COVID-19 [6]. In addition to reduced NK or T cell frequencies, functional exhaustion of these cells was demonstrated to contribute to SARS-CoV-2-mediated defective immune cell responses [6,7]. Exhaustion was first described upon long-term infection with viruses [8], but the hallmark of SARS-CoV-2 is its ability to use this inhibitory pathway in very early stages of infection. Moreover, NKG2A and PD-1 have been shown to be upregulated by SARS-CoV-2 in NK and T cells, respectively, and to be downregulated upon recovery from COVID-19, demonstrating that SARS-CoV-2 is an inducer of exhaustion [7]. Recent reports have demonstrated the resistance of mesenchymal stem cell (MSC) or ASC to SARS-CoV-2 infection [9] and the beneficial effects of healthy MSC transplantation in severe COVID-19 patients [10,11]. However, as opposed to ASC, adipocytes express the ACE2 receptor at high density, and even more when they belong to obese individuals [12], suggesting a mechanism that could account for the higher susceptibility of obese individuals to SARS-CoV-2 infection.

Obesity with a BMI of >30 Kg/m^2^ is an independent risk factor for severe forms of COVID-19, as demonstrated in several clinical departments including ours, where 25% of hospitalized patients and 35% of ICU patients below the age of 70 were found to be obese [13,14]. Indeed, obesity is associated with comorbidities such as hypertension, hypercholesterolemia, cardiovascular diseases, atherosclerosis, type 2 diabetes, chronic inflammatory diseases, and various cancers [15]. Besides the elevation of chronic inflammation markers inside adipose tissues, plasma, and/or metabolically altered organs, immune dysfunction has also been described during obesity [16]. Indeed, higher rates of infection, delayed wound healing, and vaccination failure are often described in obese individuals. The impact of obesity on immunity has been related to various mechanisms, such as metabolic alterations in T cells, acceleration of thymic aging, alteration of lymphoid organ architecture, reduction in the maintenance of memory T cells, impairment of cell-mediated immune responses, abnormal lymphoproliferative responses, and decreased cytotoxic activities related to reduced NK and CD8+ T cell frequencies, especially among elderly individuals [17,18]. Moreover, increased T cell exhaustion due to enhanced PD-1 expression has recently been reported to occur in blood T cells among obese individuals suffering from cancer [19]. As white adipose tissue (WAT) is known to initiate inflammation in obese individuals, we investigated whether increased adiposity and inflammation in obesity could result in exhaustion of WAT-infiltrating T cells. Here, we showed that obesity is associated with PD-L1 overexpression inside WAT and implicated adipose-tissue-derived mesenchymal stem cells (ASC) and IFNγ secretion in this upregulation. We also demonstrated that overexpression of PD-L1 has spread to cells present in the ASC environment, particularly to adipocytes.

## 2. Materials and Methods

### 2.1. Animals, Diet, and Experimental Design

All procedures were performed with the approval of the Regional Committee of Ethics for Animal Experiments (CECCAP), registered under number C2EA15 by the French Ministry of Higher Education and Research, in accordance with European directive 2010/63. The experimental protocol received the number 2018061411298503. Four-week-old C57Bl/6J male mice (Envigo; Gannat, France) were randomly housed, two per cage, at 21 °C with a normal light/dark cycle, free access to water, and a standard chow diet (Genestil, Royaucourt, France) for a one-week acclimatization. Next, mice were randomly assigned to four different groups (*n* = 5–6/group). One group was fed a standard diet (ST0;10% fat and 35.6% maltodextrin) and another was fed a high-fat/high-sucrose diet (HF0; 39.4% fat and 16.6% maltodextrin +16.6% sucrose, Envigo). Food was given three times a week. Body weight was recorded weekly.

### 2.2. Tissue Collection and Blood and Plasma Analyses

Before sampling, the mice were fasted for 6 h and then weighed. Blood was collected by retro-orbital sampling and mice were euthanized by cervical dislocation. Adipose tissues were quickly removed, weighed, frozen in liquid nitrogen, and stored at −80 °C. Blood glucose concentrations (Accucheck Performa glucometer; Roche Diabetes Care, Meylan, France,), plasma levels of insulin (Mouse Ultrasensitive ELISA, Eurobio, Courta-boeuf, 92973, France), leptin, and adiponectin (ELISA kits, CrystalChem Europe, Zaandam, Netherlands) were measured.

### 2.3. Isolation and Expansion of Adipose-Tissue-Derived Mesenchymal Stem Cells

Subcutaneous or visceral AT samples were isolated from residues of bariatric surgery from obese subjects (BMI > 30 kg/m^2^), or visceral surgery from lean controls with the approval of the Committee for the Protection of Human Subjects of the “Hospices Civils de Lyon” number 12/111 and with the patient’s consent, according to ethical principles for medical research involving human subjects, as described in the World Medical Association’s Helsinki Declaration. AT samples (50–100 mg) were mechanically dissected and incubated in 2 g/L of collagenase type Ia solution (Sigma Aldrich, Saint-Quentin Fallavier, France) in DMEM:F12 medium (50/50 vol/vol) (Invitrogen, Thermofisher Scientific, Waltham, MA, USA) for 40 min at 37 °C by mixing. Isolation of ASC was performed as previously reported [20]. Adipose-tissue-derived mesenchymal stem cells (ASC) were expanded in a culture medium composed of DMEM:F-12 supplemented with 10% FCS, 2 mmol/L L-glutamine, and 100 U/mL penicillin–streptomycin. Culture medium was changed by half, three times a week, during 3–4 passages before being used or stored in liquid nitrogen. The function of ASC was validated as recommended by the French Cell Therapy Society [21] and checked for expression of CD90, CD105, and CD73 molecules.

### 2.4. Isolation of Blood Mononuclear Cells (MNC)

Blood samples from the Blood Bank Center of Lyon, Lyon, 69007, France) were obtained from healthy human blood donors. MNC were separated from blood through Ficoll-Histopaque density gradient centrifugation (Sigma-Aldrich) and stored in liquid nitrogen.

### 2.5. Coculture Assays

ASC were harvested and seeded in 24-well plates (100,000 cells/well) for 24 h in RPMI supplemented with 10% FCS. Then, 24 h later, MNC were added in the presence or absence of phytohemagglutinin (PHA, 5 µg/mL, Sigma-Aldrich). ASC/MNC ratios of 1:5 were used. For mRNA analyses, the culture period was 24 h, whereas it was 48 h when conditioning media were collected. In that latter case, IL-17A neutralizing antibodies (Secukinumab, Novartis Pharma S.A.S., 92506, Rueil-Malmaison, France) at 50 μg/mL or IFNγ-neutralizing antibodies (Invitrogen, 16-7318-81) at 50 μg/mL were eventually added from the beginning of cultures. For blocking experiments, ASC/MNC cocultures were treated with PD-L1 neutralizing antibodies (R&D Systems, Minneapolis, MN, USA; AF156) at 1 or 10 μg/mL, or with irrelevant polyclonal goat IgG (sc-8828; Santa-Cruz Biotechnology/INC, Heidelberg, Germany) at 10 μg/mL, or Secukinumab at 50 μg/mL.

### 2.6. mRNA Measurements

Total RNA was extracted from cocultures using the Tri Isolation ReagentTM (Roche Diagnostics, Meylan, France) and frozen at –80 °C, and RT-qPCR was performed from reversely transcribed RNA into cDNA, as previously described [20]. mRNA was quantified relative to the housekeeping gene TBP (TATA BOX binding protein), using the mathematical method depending on ΔCT and the amplification efficiency of the transcripts, as described by Plaffl et al. [22]. The individual primer sequences used for RT-qPCR are provided in Supplementary Table S1.

### 2.7. Flow Cytometry

Fluorescein isothiocyanate (FITC)–, phycoerythrin (PE)- Allophycocyanin (APC)-, and Alexa-fluor- directly conjugated anti-human antibodies were used to stain the various cells tested: CD14 (21270146; ImmunoTools, Friesoythe, Germany), CD73 (21270734; ImmunoTools), CD8 (21270083; ImmunoTools), CD274 (12-5982-82; Invitrogen, Illkirch, France), CD279 (FAB7115G; R&D Systems, Biotechne, Abingdon, UK), and Granzyme B (IC2906G; R&D Systems). Analyses were performed using a LSR II 3 lasers cytofluorometer and Diva software (BD Biosciences, Le-Pont-de-Claix, France). For Granyme B intracellular staining, cells were permeabilized using the BD Cytofix/Cytoperm permeabilizing kit (AB_2869008; Beckton Dickinson, Le-Pont-de-Claix, France), and cells were analyzed 6 h after interaction with adipocytes. 

### 2.8. Differentiation of Obese Subcutaneous ASC into AD

Ob-ASC were differentiated into AD as previously described [20].

### 2.9. Immunohistochemistry

ATs were fixed in formaldehyde 3.7% after isolation from bariatric surgery. They were then embedded in paraffin before being cut with the microtome MICROT002 and mounted onto microscope slides for analysis, after staining with anti-human PD-L1 (R&D Systems; MAB1561) at 1:50 dilution.

### 2.10. Statistical Analysis

Statistical analyses were performed using one-way ANOVA with or without repeated measures, or mixed-effects analyses (if some values were missing), followed by post hoc Fisher’s LSD tests. In some experiments, two-tailed unpaired t-tests were used. Prism software was employed for analysis (Prism 8, GraphPad software, San Diego, CA, USA). Differences were considered as statistically significant when *p* was <0.05.

## 3. Results

### 3.1. PD-L1 and/or PD-1 Are Overexpressed in the WAT of Obese Mice

**Figure 1:**
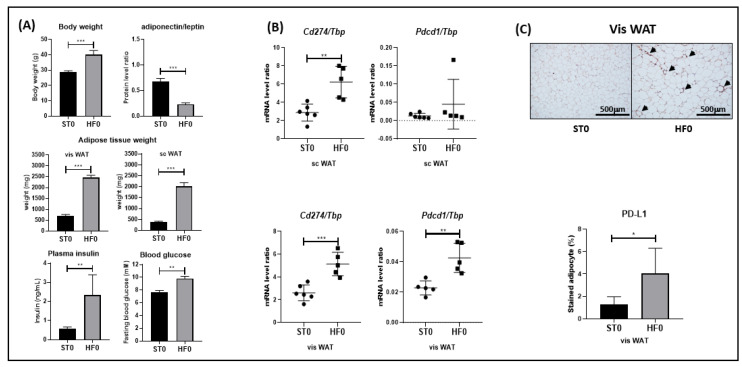
PD-L1 and/or PD-1 are overexpressed in the WAT of obese mice. C57BL/6 mice were fed with a normal or hypercaloric diet for 16 weeks (STO versus HFO). (**A**) Body and adipose tissue weight were measured, together with plasma insulin and blood glucose, after sacrifice. (**A**,**B**) serum adiponectin and leptin were measured by ELISA. *Cd274(Pdl1)* and *Pdcd1(Pd1)* mRNA were extracted from subcutaneous (sc) or visceral AT and their levels measured by RT-PCR. (**C**) Immunohistochemistry staining of PD-L1 in vis WAT. Results are expressed as a ratio relative to the *Tbp* housekeeping gene. (**A**,**C**) Black or grey histograms correspond to STO, or HFO mice, respectively. (**B**) Black circles, or black squares correspond to STO, or HFO mice, respectively. (**C**) Black triangles correspond to positively stained PD-L1 molecules. Two-tailed unpaired *t*-tests were used to prove the statistical significance of the results when *p* was <0.05. ***, **, * represents a *p*-value of <0.001, <0.01, <0.05 respectively. Data are the mean ± SD of *n* = 6, or 5 for STO or HFO, respectively.

Whereas blood T cells from obese individuals or mice have been shown to overexpress the PD-1 checkpoint [19], here we investigated the possibility that WAT-infiltrating T cells could also express markers of exhaustion among the obese. With this goal in mind, we used a high-fat-diet-induced obesity (DIO) mouse model, wherein postweaning C57BL/6 male mice were fed either a control (chow) or hypercaloric diet for 16 weeks, and we measured the mRNA expression levels of PD-L1/PD-1exhaustion markers. As shown in Figure 1A, the efficiency of the hypercaloric diet was assessed through (i) increased body weight and white adipose tissue mass; (ii) metabolic consequences, such as increased fasting plasma insulin and blood glucose; and (iii) adipokine disturbances, such as decreased adiponectin over leptin protein levels (Figure 1A). In addition, PD-L1 was overexpressed at the mRNA level in both the visceral (vis) and subcutaneous (sc) WAT of obese, in comparison with lean mice (Figure 1B). However, increased PD-1 expression was observed in the vis but not sc WAT of mice fed with the hypercaloric diet (Figure 1B), which suggested more pronounced inhibition of T cell functions in vis. To support these results, immunochemistry was used to stain obese or lean visceral WAT for PD-L1. As shown in Figure 1C, a significant increase in PD-L1 expression was observed in the vis WAT of obese mice.

### 3.2. Inflammation-Mediated by Adipose-Tissue-Derived Mesenchymal Stem Cells from Obese Individuals Contributes to PD-L1 Upregulation in Cocultures with Mononuclear Cells

**Figure 2:**
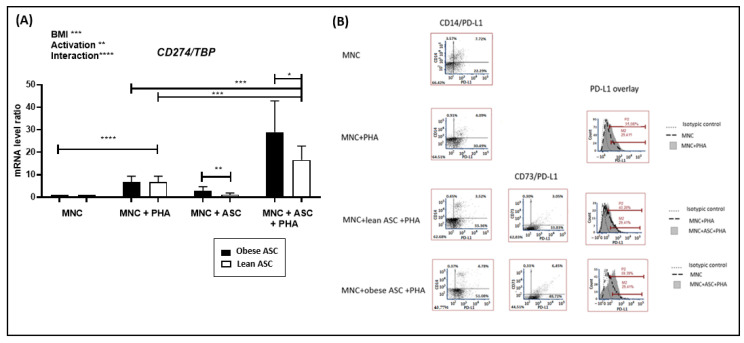
Inflammation, mediated by adipose-tissue-derived mesenchymal stem cells (ASC) from obese individuals, contributes to PD-L1 upregulation in cocultures with mononuclear immune cells. MNC were activated by PHA in the presence or absence of ob-ASC or lean ASC. (**A**) *CD274* (*PDL1)* mRNA expression levels were measured by RT-qPCR. Results are expressed as a ratio relative to the *TBP* housekeeping gene. Two-way ANOVA with Fisher’s LSD comparison tests were used. Data are the mean ± SD of *n* = 4. ****, ***, **, and * represent a *p*-value <0.0001, <0.001, <0.01, or <0.05, respectively. (**B**) Membrane expression of PD-L1 was measured by flow cytometry in cells stained for CD14 or CD73. Results are representative of two experiments with two different ASC.

Having previously demonstrated that adipose-tissue-derived mesenchymal stem cells (ASC) from obese individuals (ob-ASC), rather than from lean individuals, can promote inflammation through Th17 cell and monocyte cell activation [23], we then investigated whether inflammation-mediated by ob-ASC could play a role in upregulating PD-L1 and/or PD-1 expression using a human in vitro coculture model. As shown in Figure 2A, phytohemagglutinin A (PHA) activation of mononuclear cells (MNC) enhanced *PDL1* mRNA expression levels, but the co-presence of ASC resulted in potentiating the expression of *PDL1* mRNA, whether ASC were issued from lean or obese donors. However, ob-ASC upregulated *PDL1* mRNA expression at a statistically significant higher level than lean ASC. In addition, a two-way ANOVA test demonstrated (i) that both the body mass index (BMI) and the activation status were statistically significant independent factors, (ii) as well as the interaction between these two factors (Figure 2A). Then, to validate those results at the protein level and investigate whether increased PD-L1 levels occurred in MNC or ASC themselves, we co-stained monocytes or ASC with the CD14 or CD73 marker, respectively, and the PD-L1 molecules. As shown in Figure 2B, flow cytofluorometry supported the mRNA data. Indeed, an increased surface expression of PD-L1 occurred, following PHA activation of MNC (i.e., 29.4–35.1% of positive PD-L1 cells), which was potentiated by the presence of ob-ASC (29.4–59.4%), but with a weaker potentiating effect in the presence of lean ASC (29.4–43.2%). CD14 labelling showed that PD-L1 was expressed at the basal level in monocytes with approximately 2/3 of the CD14 positive cells expressing both CD14 and PD-L1 (i.e., 7.7% of cells were doubly positive for CD14 and PD-L1, versus 3.6% singly positive for CD14). However, following 48 h of activation with PHA, almost the whole population of CD14-positive cells became doubly positive for PD-L1, even though their ratio diminished from 7.7% to 4.05% of total cells, probably due to the proliferation of PHA-activated T cells. The presence of obese or lean ASC did not induce any upregulation of the surface expression level of PD-L1 in monocytes. However, it resulted in the upregulation of the intensity of CD14 fluorescence, suggesting the transition of monocytes towards a population of “classical monocytes”, as previously described [24]. Moreover, PD-L1 expression in CD73+ ob-ASC was higher than in lean ASC (i.e., from 3.0% to 6.4%). However, the prominent population of cells in which PD-L1 expression was upregulated was CD73-negative (i.e., from 33.8% to 48.7%). This latter population was likely to correspond to activated T lymphocytes, as T cells were the most preponderant cells in those cocultures, on the one hand, and are known to express PD-L1 expression following antigenic stimulation, on the other hand [25,26]. Altogether, those results demonstrated that PD-L1 expression is upregulated following activation with PHA at both the transcriptional and protein levels, and was considerably amplified by the presence of ob-ASC.

### 3.3. Activation of T Cells Upregulates PD-1 Expression but Is Not Influenced by the Presence of Adipose-Derived Stem Cells (ASC)

**Figure 3:**
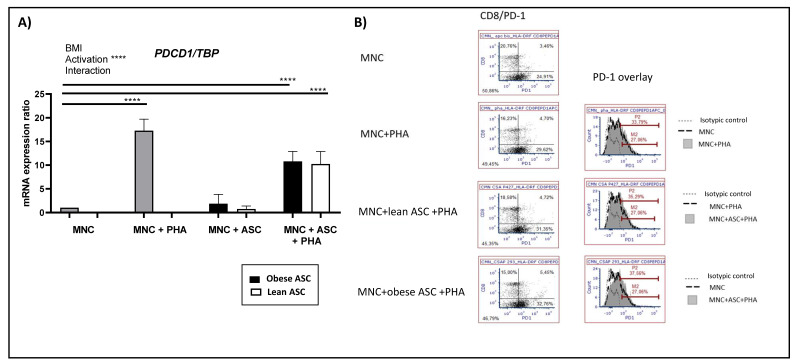
Activation of T cells upregulates PD-1 expression but is not influenced by ASC. MNC were activated by PHA in the presence or absence of ob-ASC or lean ASC. (**A**) *PDCD1 (PD1)* mRNA expression levels were measured by RT-qPCR. Results were expressed as a ratio relative to the *TBP* housekeeping gene. Two-way ANOVA with Fisher’s LSD comparison tests were used. Data are the mean ± SD of *n* = 4. **** represents a *p*-value <0.0001. (**B**) Membrane expression of PD-1 was measured by flow cytometry in cells also stained for CD8. Results are representative of two experiments with two different ASC.

Because PD-1 needs to be linked by PD-L1 to exert its inhibitory effect on T cells, we then investigated whether the presence of obese ASC could also upregulate its expression. As shown in Figure 3A, PHA activation of MNC increased the levels of PD1 mRNA expression, but the presence of obese or lean ASC did not further influence its expression. Supporting these results, and as shown in Figure 3B, PD-1 surface expression was enhanced in PHA-activated MNC (i.e., from 27% to 33.79%). Those levels barely increased from 33.79% to 35.29% or 37.56% in the presence of lean or ob-ASC, respectively. The levels of CD8+ doubly positive cells for PD-1 increased, i.e., from 3.5% in the resting state to 4.7% with PHA, and were barely increased in the presence of ob-ASC from 4.7% to 5.45%. Therefore, these results suggested that PD-1 is likely to be a marker of activation, with obese ASC influencing PD-L1, but not—or only slightly—PD-1 expression.

### 3.4. PD-L1 Blockade Partially Restores Th1 Cell Secretion and Improves Ob-ASC Mediated Th17 Cell Activation, without Affecting Pro-Inflammatory Cytokine Secretion by Accessory Cells

**Figure 4:**
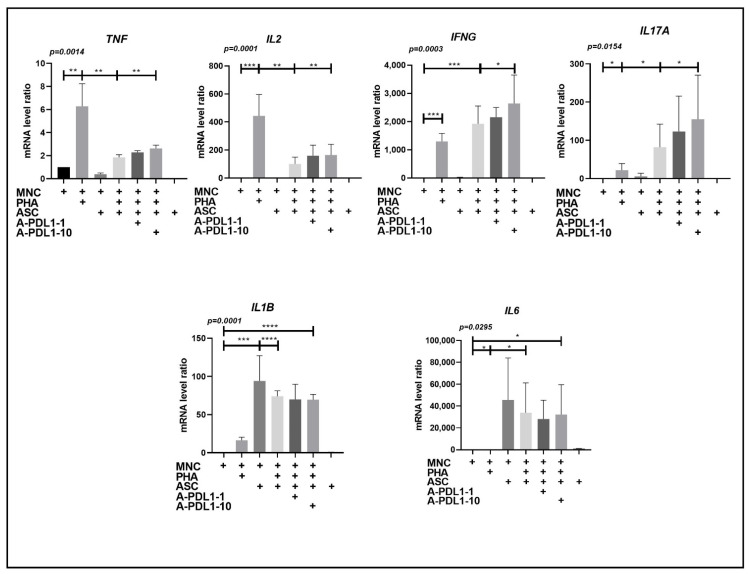
PD-L1 blockade partially restores Th1 cell secretion and improves ob-ASC mediated Th17 cell activation without any effect on pro-inflammatory cytokine secretion by accessory cells. MNC were activated by PHA in the presence or absence of ob-ASC and anti-PD-L1mAbs at concentrations of 1 or 10 μg/mL. Cytokine mRNA expression levels were measured by RT-qPCR. Results were expressed as a ratio relative to the *TBP* housekeeping gene. The one-way ANOVA with Fisher’s LSD comparison tests were used. Data are the mean ± SD of *n* = 4. The *p*-value shown in the figure is from an ANOVA test, whereas ****, ***, **, or * represents *p*-values of <0.0001, <0.001, <0.01, <0.05 respectively, from Fisher’s LSD tests.

To then analyze the consequences of PD-L1 overexpression, we measured the effects of PD-L1 blockade on the pro-inflammatory cytokine expression. As previously reported [23] and as shown in Figure 4, the presence of ob-ASC resulted in increasing IL-17A, IL-1Β, and IL-6, together with decreasing TNFα and IL-2 mRNA expression levels, as compared with PHA-activated MNC. The modification of the cytokine pattern has been previously reported by us to be related to (i) the immunomodulatory effect of ASC on Th1 responses, (ii) the promotion by ob-ASC of Th17 double positive cells, secreting both IL-17 and IFNγ, and (iii) the activation of pro-inflammatory secretion by accessory cells such as monocytes and ob-ASC [23]. The addition of neutralizing anti-PD-L1 mAbs in those cocultures resulted in the partial restoration of Th1 cell responses, as assessed by increased TNFα and IL-2 mRNA expression levels. An increase in IL-17A and IFNγ mRNA expression by Th17 cells was also observed. However, *IL1Β* and *IL6* mRNA expression levels were not modified, thus demonstrating a specific PD-L1 inhibitory effect on T cell functions. The specificity of the anti-PD-L1 effects was supported by further experiments comparing IL-17 and PD-L1 blockade (Figure S1). 

### 3.5. Ob-ASC-Mediated Inflammation Results in Increasing PD-L1 Expression in Freshly Differentiated Adipocytes

**Figure 5:**
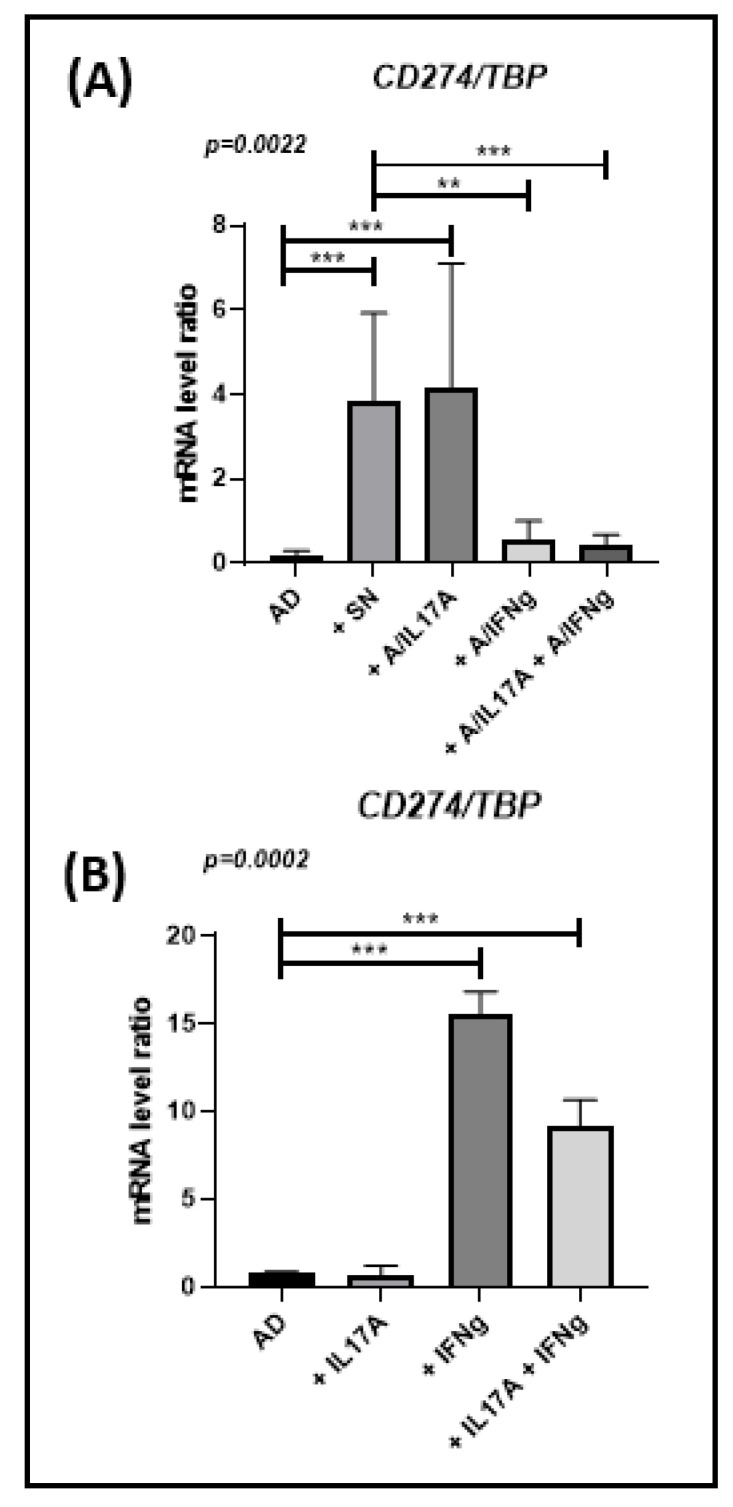
Ob-ASC mediated inflammation results in increasing *PDL1* mRNA expression in freshly differentiated adipocytes. Adipocytes were differentiated from ob-ASC in the presence or absence of conditioned media (CM) collected from activated ob-ASC/MNC cocultures, with or without anti-IL-17A, anti-IFNγ, or both mAbs (**A**), or the presence of IFNγ, IL-17A, or both cytokines (**B**). *CD274* (*PDL1)* mRNA expression levels were measured by RT-qPCR. Results were expressed as a ratio relative to the *TBP* housekeeping gene. One-way ANOVA with Fisher’s LSD comparison tests were used. (*n* = 4 in (**A**), or 2 in (**B**)). The *p*-value shown in the figure are those of ANOVA tests, whereas ***, ** represent *p*-values <0.001 or <0.01, respectively, of Fisher’s LSD tests.

Because the micro-environment plays an important role in the propagation of inflammation in WAT, we then asked whether conditioned media (CM) collected from ob-ASC and MNC cocultures could propagate exhaustion. Therefore, we cultured freshly differentiated adipocytes from ob-ASC with CM harvested from PHA-activated cocultures. We observed a strong upregulation of the expression of *PDL1* mRNA, up to almost 4-fold. Having previously demonstrated that Th17 cells play an important role in the propagation of inflammation in this coculture model [20], we then asked whether cytokines secreted by those T cells, i.e., IL-17A and/or IFNγ, could play a role in *PDL1* mRNA overexpression in adipocytes. Therefore, neutralizing antibodies directed against IFNγ or IL-17A were added or not to these cocultures. The results clearly showed that CM-mediated *PDL1* mRNA overexpression was dependent on IFNγ secretion only, since blockade of IFNγ but not IL-17A prevented CM effects on *PDL1* mRNA overexpression (Figure 5A). To validate these results, we reciprocally added IFNγ, or IL-17A recombinant protein to adipocytes and observed that IFNγ, but not IL-17A, significantly upregulated *PDL1* mRNA expression (Figure 5B).

## 4. Discussion

Brown adipocytes and differentiated 3T3 cell lines have been shown to express PD-L1 [27,28]. Additionally, we demonstrated that white adipocyte precursors can also overexpress PD-L1 following interaction with MNC, especially when they were collected from obese individuals (Figure 2). Interestingly, we demonstrated that inflammation mediated by ob-ASC and MNC cocultures spread PD-L1 overexpression to cells present in their environment, especially adipocytes, under the influence of IFNγ secretion (Figure 5). A role for IFNγ in the induction of exhaustion has already been reported by others, particularly in a tumoral model [4,29], thus demonstrating a regulatory role for IFNγ, in addition to its role in activating effectors such as macrophages or other accessory cells. Moreover, our data suggested that the negative modulation of Th1 cells that occurred in the presence of ob-ASC was partly related to IFNγ-mediated PD-L1 overexpression, as the PD-L1 blockade partially restored Th1 cell cytokine secretion (Figure 4).

IL-2 is a cytokine that plays a major role in the maturation of precytotoxic T cells into cytotoxic T cells (CTL) in order to allow them to subsequently exert their cytotoxic function [30]. We have previously reported that the presence of mesenchymal stem cells inhibit pre-CTL differentiation into CTL effectors, which is restored by the addition of IL-2 [31]. Therefore, our results suggest that ob-ASC may inhibit the maturation of pre-CTL into CTL effectors and subsequent CTL function through IL-2 negative modulation. Moreover, PD-L1 overexpression may partly account for this inhibition, as demonstrated by the positive effects of the PD-L1 blockade on *IL2* mRNA expression (Figure 4). Of interest, CTL activity was reduced in the presence of obese adipocytes in comparison with lean adipocytes, as demonstrated by granzyme B intracellular staining (Figure S2), thus suggesting that ASC-mediated spreading of PD-L1 overexpression towards obese adipocytes may inhibit obese adipocytes’ ability to activate CTL effectors.

PD-1 was also found to be upregulated following activation of MNC with PHA (Figure 3). However, this upregulation was not potentiated by the presence of ASC, suggesting that PD-1 is a marker of activation, in agreement with several studies reporting a regulatory function of PD-1 or other immune checkpoints, notably CTLA-4 [32,33,34,35]. Indeed, PD-1 was demonstrated to be transiently expressed during acute viral infections in order to regulate T cell functions. However, in chronic infection with LCMV or HIV, high levels of PD-1 were shown to be sustained over time and to contribute to T cell exhaustion [36,37]. In our coculture model, we were not able to compare acute and chronic stimulation. However, when mice were fed a hypercaloric regimen for 16 weeks, which corresponded to a chronic situation, we observed an upregulation of PD-1 along with PD-L1 in the vis WAT of obese mice in comparison to the vis WAT of lean mice (Figure 1). However, in the sc WAT of obese mice, PD-L1 but not PD-1 was upregulated, probably due to the higher levels of pro-inflammatory cytokine secretion known to be present in vis WAT [38].

Altogether, these data lead us to suggest that increased expression of PD-L1 on adipocytes and of PD-1 on T cells may trigger exhaustion and inhibit T cell functions, in particular IL-2 secretion and subsequent CTL function. This could be of high importance in situations where viruses infiltrate WAT. Infection with SARS-CoV-2 could represent one such situation, as AT is known to express ACE2, a receptor for the spike protein of SARS-CoV-2 [12]. Interestingly enough, the expression of ACE2 has been shown to be upregulated in the epicardial fat of obese versus lean mice [39]. Therefore, increased exhaustion of T cells in the inflammatory environment present in obese visceral WAT could help SARS-CoV-2 to reside inside it, which may lead us to consider fat as a reservoir for this virus. It would then become easy for the virus to invade and spread in proximal tissues or organs, as suggested by Nishimura et al. for the H5N1 virus [40]. In that case, anti-PD-1 or anti-PD-L1 biologics could become a promising alternative approach to help obese individuals achieve a better immune response against the severe form of COVID-19, as shown in the graphical abstract. Accordingly, when PD-L1 was neutralized with specific antibodies, we observed a partial restoration of cytokines expressed by Th1 cells, notably IL-2, which plays a critical role in precytolytic (CTL) differentiation into CTL effectors (Figure 4). TNFα, IL-17A, and IFNγ also increased, demonstrating the inhibitory effect of PD-L1 on T cell function whatever the T cell subset. Moreover, IL-1β and IL-6 pro-inflammatory cytokines, which were shown to be secreted by monocytes and ob-ASC in our model [17], did not increase after PD-L1 blockade, thus demonstrating the specificity of the PD-L1 blockade.

## 5. Limitations of the Study

This study was performed first in vivo in animals and then in in vitro coculture assays with human ASC and MNC. These assays have been used to investigate the mechanisms leading to increased expression of immune checkpoints, as observed in vivo. Although these coculture assays have been previously used to demonstrate the role of ASC, Th17 cells, and monocytes in initiating inflammation among AT from obese individuals [23], they have some limitations relative to the cell isolation and 2D culture processes and thus do not exactly correspond to the in vivo situation. Then, we demonstrated that inflammation may lead to exhaustion of T cells following interaction of MNC with ASC relative t increased expression of immune checkpoints, and suggested, in Section 4, that T cell exhaustion in WAT could contribute to the higher susceptibility of obese individuals to viruses infecting WAT, as seen during the COVID-19 pandemic. However, we did not directly test this hypothesis in the present study, such as by investigating the cytolytic activity against adipocytes previously infected by viruses, and notably SARS-CoV-2, in the presence or absence of anti-PD-L1. Indeed, such investigation would require additional experiments with specific SARS-CoV-2-infected adipose tissue, which is not available in our laboratory due to regulatory aspects.

## 6. Conclusions

In this study, we analyzed the impact of obesity on immune checkpoint expression in WAT, using a mouse model of diet-induced obesity. We observed that PD-L1 is overexpressed in WAT of obese mice, and is associated with increased expression of PD-1 in visceral WAT as compared with lean mice. Using human in vitro cocultures with ASC from obese individuals and MNC, we also observed an overexpression of PD-L1 with respect to cocultures with ASC from nonobese individuals, Moreover, conditioned media harvested from these cocultures enhanced PD-L1 expression in freshly differentiated adipocytes, depending on the presence of IFNγ. As adipocytes differentiated from obese ASC impaired cytotoxic activity, our results suggest that PD-L1 overexpression may occur in the visceral WAT of obese individuals under IFNγ secretion and may lead to T cell dysfunction, notably decreased cytolytic activity. Such a mechanism could shed some light on why adipose tissue-infiltrating viruses such as SARS-CoV-2 can worsen disease in obese individuals.

## Data Availability

The data that support the findings of this study are available within the article.

## Supplementary Materials

The following are available online at https://www.mdpi.com/article/10.3390/cells10102645/s1, Figure S1: Specificity of PD-L1 blockade, as compared with IL-17A blockade, Figure S2: CTL activity is reduced in the presence of obese versus lean ASC, Table S1: List of primers used for RT-QPCR in the study.
