# Supplementary material for: Adipose-Tissue-Derived Mesenchymal Stem Cells Mediate PD-L1 Overexpression in the White Adipose Tissue of Obese Individuals, Resulting in T Cell Dysfunction
Source: Cells. 2021 Oct 3;10(10):2645. doi: 10.3390/cells10102645 (PMC8534339; doi:10.3390/cells10102645)
Supplement: Supplementary file 1 [file cells-10-02645-s001.zip › cells-1337298-supplementary.pdf]

Adipose tissue-derived mesenchymal stem cells mediate PD-L1 overexpression in white adipose tissues of obese individuals, resulting in T-cell dysfunction

Assia Eljaafari[1,2,,3]*, Julien Pestel[1,2], Brigitte Le Magueresse-Battistoni[1,2], Stephanie Chanon[1,2],

Julia Watson[1,2], Maud Robert[1,2, 4], Emmanuel Disse[1,2,5,] Hubert Vidal[1,2]

Supplementary Informations :

Supplementary Figure S1

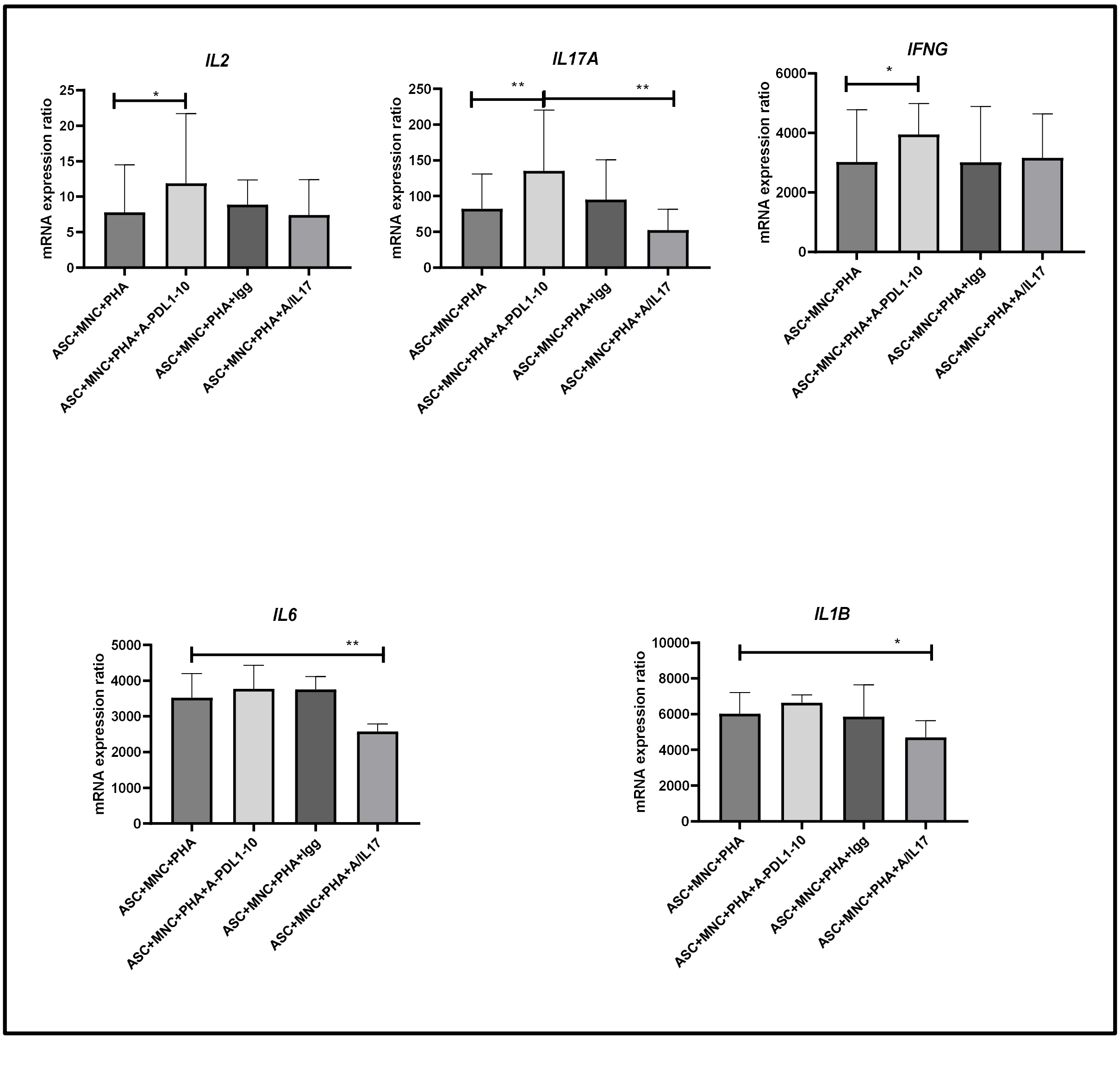

Figure S1*: Specificity of PD-L1 blockade, as compared with IL-17A blockade.* MNC were activated by PHA in the presence of ob-ASC and anti-PD-L1mAbs (R&D Systems, AF156,10

μg/ml), anti-IL17A Ab (Secukinumab, Novartis Diagnostics, Pharma S.A.S., 92506, Rueil-Malmaison, France; 50μg/ml) or irrelevant polyclonal goat IgG (Santa-Cruz Biotechnology/INC, Europe; sc-8828, 10μg/ml). Cytokine mRNA expression levels were measured by RT-qPCR, as mentioned in the Materials and Methods section. Results were expressed as a ratio relative to the *TBP* house-keeping gene. Non parametric *t* tests were used. Data are the mean ± SD of n=4. **,*represent a p value <0.01, or <0.05, respectively.

Supplementary Figure S2

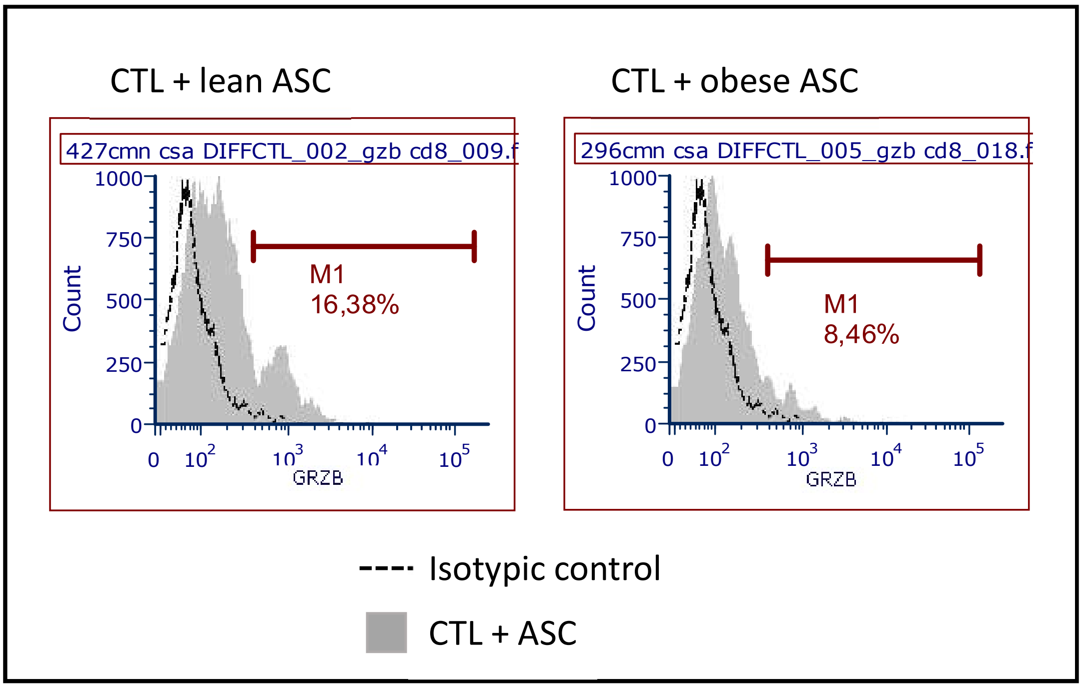

Figure S2: ***CTL activity is reduced in the presence of obese versus lean ASC,***

CTL were obtained following co-culture with obese or lean ASC in the presence of PHA for 72 hours, washed away from PHA, rested for one day and and expanded in the presence of IL-2 (R&D Systems, Minneapolis, MN, USA); 20UI/ml) for 8 additional days. In the meanwhile, lean or obese adipocytes were differentiated from the same ASC used in the co-culture experiments for 8 days, using the protocol described in the Materials and Section of the paper. After 12 days, cells were then restimulated with the differentiated adipocytes for 6 hours before being permeabilized and stained for granzyme B. Intra-cellular secretion of the enzyme was measured by cytofluorometry. This figure is representative of 2 experiments using different ASC

Table S1 ***List of primers used in RT-qPCR***

Human primers		
**Gene**	**Forward primer**	**Reverse primer**
TBP	AGACCATTGCACTTCGTGCC	CCTGTGCACACCATTTTCCC
CD274 (PDL1)	CTGCAGGGCATTCCAGAAAG	GTTCAGCAAATGCCAGTAGG
PDCD1	CCCAAGGCGCAGATCAA	GCACTTCTGCCCTTCTCTCTGT
TNF	AGCCCATGTTGTAGCAAACC	GAGGTACAGGCCCTCTGATG
IL2	CAAACTCACCAGGATGCTCA	GCACTTCCTCCAGAGGTTTG
IFNG	GATGACCAGAGCATCCAAAAG	CATGTATTGCTTTGCGTTG
IL17A	ACCAATCCCAAAAGGTCCTC	TGGTAGTCCACGTTCCCATC
IL1B	GGCAATGAGGATGACTTGTT	TGTAGTGGTGGTCGGAGATT
IL6	AGCCCTGAGAAAGGAGACATGTAACAAG	TTCTGCAGGAACTGGATCAGGACTTT

Murine primers		
**Gene**	**Forward primer**	**Reverse primer**
Tbp	TGGTGTGCACAGGAGCCAAG	TTCACATCACAGCTCCCCAC
Cd274 (Pdl1)	AATGTGACCAGCAGTCTGAG	AAGCACCCAGTGAGTCCTGT
Pdcd1	CTAGCTGTCTTCTGCTCAAC	GGAAGTCCAGCTCCTCATAG
